# A Novel D-Psicose 3-Epimerase from Halophilic, Anaerobic *Iocasia fonsfrigidae* and Its Application in Coconut Water

**DOI:** 10.3390/ijms24076394

**Published:** 2023-03-29

**Authors:** Shinta Wulansari, Sobroney Heng, Prattana Ketbot, Sirilak Baramee, Rattiya Waeonukul, Patthra Pason, Khanok Ratanakhanokchai, Ayaka Uke, Akihiko Kosugi, Chakrit Tachaapaikoon

**Affiliations:** 1 Division of Biochemical Technology, School of Bioresources and Technology, King Mongkut’s University of Technology Thonburi, Bangkok 10150, Thailand; 2 Excellent Center of Enzyme Technology and Microbial Utilization, Pilot Plant Development and Training Institute, King Mongkut’s University of Technology Thonburi, Bangkok 10150, Thailand; 3 Biological Resources and Post-Harvest Division, International Research Center for Agricultural Sciences (JIRCAS), 1-1 Ohwashi, Tsukuba 305-8686, Japan

**Keywords:** coconut water, halophilic enzyme, *Iocasia fonsfrigidae*, D-psicose, D-psicose 3-epimerase, rare sugar

## Abstract

D-Psicose is a rare, low-calorie sugar that is found in limited quantities in national products. Recently, D-psicose has gained considerable attention due to its potential applications in the food, nutraceutical, and pharmaceutical industries. In this study, a novel D-psicose 3-epimerase (a group of ketose 3-epimerase) from an extremely halophilic, anaerobic bacterium, *Iocasia fonsfrigidae* strain SP3-1 (IfDPEase), was cloned, expressed in *Escherichia coli*, and characterized. Unlike other ketose 3-epimerase members, IfDPEase shows reversible epimerization only for D-fructose and D-psicose at the C-3 position but not for D-tagatose, most likely because the Gly218 and Cys6 at the substrate-binding subsites of IfDPEase, which are involved in interactions at the O-1 and O-6 positions of D-fructose, respectively, differ from those of other 3-epimerases. Under optimum conditions (5 µM IfDPEase, 1 mM Mn^2+^, 50 °C, and pH 7.5), 36.1% of D-psicose was obtained from 10 mg/mL D-fructose. The IfDPEase is highly active against D-fructose under NaCl concentrations of up to 500 mM, possibly due to the excessive negative charges of acidic amino acid residues (aspartic and glutamic acids), which are localized on the surface of the halophilic enzyme. These negative charges may protect the enzyme from Na^+^ ions from the environment and result in the lowest pI value compared to those of other 3-epimerase members. Moreover, without adjusting any ingredients, IfDPEase could improve coconut water quality by converting D-fructose into D-psicose with a yield of 26.8%. Therefore, IfDPEase is an attractive alternative to enhancing the quality of fructose-containing foods.

## 1. Introduction

Rare sugars have received considerable attention for their potential application in a wide range of biotechnological process. One of the most popular rare sugars is D-psicose (D-ribo-2-hexulose or D-allulose), an epimer of D-fructose at the C-3 position. It has been approved as generally recognized as safe (GRAS) by the United States Food and Drug Administration [1]. Due to its wide range of positive physiological and biological properties, D-psicose has gained increased attention and is commercially produced as an ingredient in a variety of food, dietary supplements, and beverages in Japan, Korea, the United States, and the United Kingdom. In addition, D-psicose has several beneficial effects on human health, such as anti-atherosclerosis, anti-diabetic, anti-inflammatory, anti-obesity, and anti-oxidation effects. It also has neuroprotective functions and can reduce postprandial blood glucose levels [1,2].

Ketose 3-epimerases, which can efficiently catalyze the C-3 epimerization of free keto sugars, can be classified into three groups, D-tagatose 3-epimerase (DTEase), D-psicose 3-epimerase (DPEase), and D-fructose 3-epimerase (DFEase), based on the principle of maximum substrate specificity [3]. These three 3-epimerases are related in terms of amino acid sequence, molecular weight, optimum pH, optimum temperature, metal ion requirement, three-dimensional structure, conserved residues in the substrate binding and catalytic sites, and catalytic mechanism [2,3] Moreover, these three groups of enzymes show broad substrate specificity by catalyzing reversible epimerization reactions at the C3 position between D-psicose and D-fructose and between D-tagatose and D-sorbose [1,4].

The D-psicose can be synthesized through the C-3 epimerization of D-fructose by DPEases (EC 5.1.3.30) with ketone group conversion [5]. Although several ketose 3-epimerases from numerous microorganisms have been reported, there have been no reports of DPEase and other ketose 3-epimerases from halophilic microorganisms. Furthermore, halobacterium enzymes are highly stable and active under high salt concentrations, and several enzymes obtained from these non-pathogenic and safe bacteria can be used in a wide variety of biotechnological applications [6,7].

Coconut water, a natural, nutritious, healthy, and therapeutic drink from coconut palm trees, is an ancient tropical beverage that has continued to gain popularity in international markets as a thirst-quenching drink, sport beverage, and natural, healthy drink [8]. Coconut water has many biological properties, such as anti-thrombotic activities, blood pressure control effects, and diarrhea control activity. However, coconut water is high in sugar, especially D-fructose, which can have negative health effects, such as obesity and diabetes [9].

*Iocasia fonsfrigidae* strain SP3-1 was isolated from a salt evaporation pond as an extremely halophilic, anaerobic bacterium that grows well in a medium containing 20% (*w*/*v*) NaCl. The whole genome sequence of this bacterium revealed the presence of a possible DPEase gene [10] for which the amino acid sequence of the DPEase from *I. fonsfrigidae* strain SP3-1 (IfDPEase) has a low similarity to other ketose 3-epimerase members. Therefore, we speculated that the IfDPEase may represent a new function in the ketose 3-epimerase family. In this study, the *IfDPEase* gene was cloned, expressed in *Escherichia coli*, and the purified recombinant enzyme was characterized. Moreover, to increase the quality of coconut water, the conversion of D-fructose in coconut water into D-psicose by this enzyme was investigated.

## 2. Results

### 2.1. Amino Acid Sequence Analysis

The gene encoding IfDPEase, which corresponds to the gene position at 533 under locus tag “D7D81_07110” (GenBank ID: OP035404), was selected for this study. This gene was amplified and ligated to the plasmid vector pET-22b(+), yielding *IfDPEase*, which was introduced into *E. coli* DH5α. The positive transformant that expressed IfDPEase activity was confirmed by DNA sequencing. The IfDPEase showed a low sequence similarity (approximately 19–25%) to other ketose 3-epimerase members from *Bacillus* sp. KCTC 13219, *C. cellulolyticum* H10, *Agrobacterium* sp. ATCC 31749, *Ruminococcus* sp., *A. tumefaciens*, *R. sphaeroides*, *T. primitia* ZAS-1, *C. scindens* 35704, *B. produca*, *P. cichorii*, *Desmospora* sp. 8473, *C. fortuita*, *Clostridium bolteae*, and *Dorea* sp. CAG317 (Table 1).

**Figure 1:**
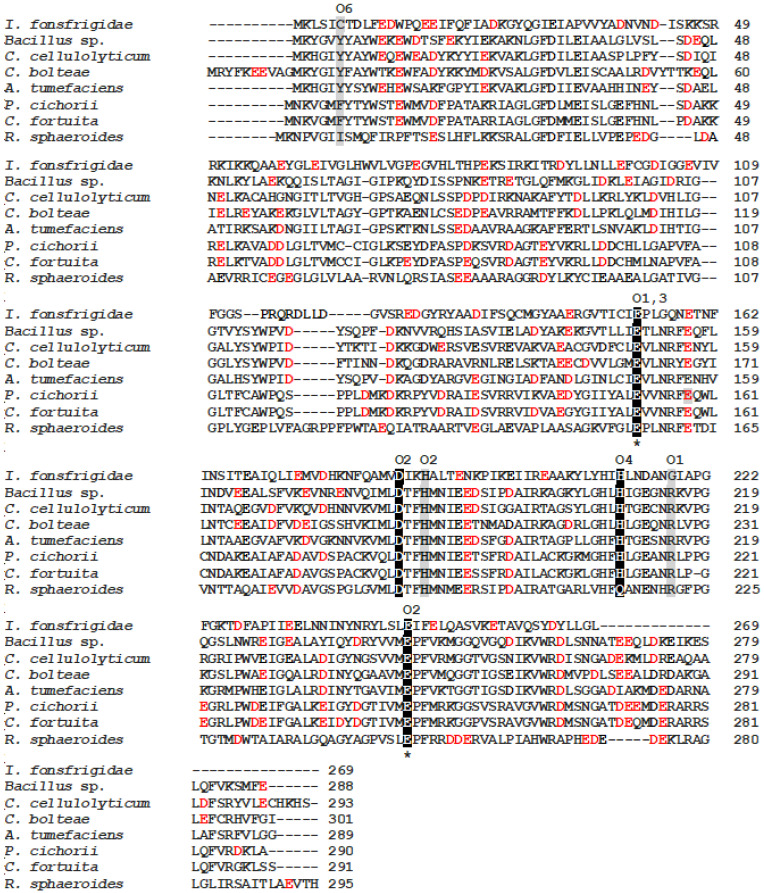
Amino acid sequence alignment of IfDPEase compared to other ketose 3-epimerase members. The substrate-binding subsite residues are shown in gray, the metal-coordinating site residues are marked in black, and the catalytic site residue is marked in black with an asterisk. Red letters represent acidic amino acids on the surface of ketose 3-epimerase structures. Sequence for DPEase from *I. fonsfrigidae* strain SP3-1 (GenBank accession no: OP035404) was aligned with those of DPEases from *Bacillus* sp. KCTC 13219 (KYG89858.1), *C. cellulolyticum* H10 (ACL75304), *Clostridium bolteae* (EDP19602), *Agrobacterium tumefaciens* (AAK88700.1); DTEases from *P. cichorii* (O50580) and *Caballeronia fortuita* (WP_061137998.1); and DFEase from *Rhodobacter sphaeroides* (ACO59490.1). Alignment was constructed with ClustalW program https://www.ebi.ac.uk/Tools/msa/clustalo/ (accessed on 10 September 2022).

The amino acid sequence of IfDPEase was aligned with other characterized ketose 3-epimerase members from the NCBI database, and the multiple sequence alignment of these enzymes was analyzed https://www.ebi.ac.uk/Tools/msa/clustalo/ (accessed on 10 September 2022). The 3D structure and active site of the DPEase from *C. cellulolyticum* H10 are already known [11]. As a result, the IfDPEase enzyme exhibited conserved amino acids of the catalytic site residues (Glu153 and Glu247) and divalent metal-coordinating site residues (Glu153, Asp186, His212, and Glu247) with other related ketose 3-epimerase members (Figure 1, Table 2). Moreover, there are six substrate-binding subsites of the DPEase involved in the interaction with the O-1 to O-6 positions of D-fructose [11]. Although most of the amino acid residues in the substrate-binding subsites of IfDPEase are identical to other ketose 3-epimerase members, the substrate-binding subsites of IfDPEase interacting with the O-1, O-4, and O-6 positions of D-fructose showed different amino acid residues comparted to other ketose 3-epimerases (Table 2).

The substrate-binding subsite of IfDPEase that interacts with the O-1 position of D-fructose is glycine, whereas the other ketose 3-epimerase member is arginine. The substrate-binding subsite of the DPEase member, including IfDPEase interaction with the O-4 position of D-fructose, is histidine, while the DFEase member is glutamine. On the other hand, the substrate-binding subsite interaction of IfDPEase with the O-6 position of D-fructose is cystine, and the other DPEase member is tyrosine, the DTEase member is phenylalanine, and the DFEase member is isoleucine (Table 2). Furthermore, histidine is one of the metal-coordinating sites of all DPEase and DTEase members, but for the DFEase member, it is glutamine.

As is shown in Figure S1, a phylogenetic tree analysis revealed that IfDPEase was individually separated from other ketose 3-epimerase members, including the DPEases from *Bacillus* sp. KCTC 13219, *Desmospora* sp. 8473, *T. primitia* ZAS-1, *C. bolteae*, *Ruminococcus* sp., *C. cellulolyticum* H10, *C. scindens* 35704, *Dorea* sp. CAG317, *A. tumefaciens*, *Agrobacterium* sp. ATCC 31749, and *B. produca*, as well as DTEases from *P. cichorii* and *C. fortuita*, and DFEase from *R. sphaeroides*. However, IfDPEase revealed the most evolutionary relationship with *Bacillus* sp. KCTC 13219 and *Desmospora* sp. 8473 DPEases.

### 2.2. Protein Expression and Purification and Allergen Analysis

The recombinant DPEase from *I. fonsfrigidae* strain SP3-1 was successfully expressed in *E. coli* BL21 (DE3). The recombinant protein, which contained a fusing protein with the C-terminal histidine tag, was purified with a HisTrap™ FF column. The purity of the recombinant protein was analyzed by sodium dodecyl sulfate polyacrylamide gel electrophoresis (SDS-PAGE). It showed a single band with a molecular weight of approximately 30 kDa (Figure S2A), corresponding to the predicted value (30.34 kDa). However, the native-PAGE revealed that this enzyme had a dimeric form (Figure S2B). Due to the partial absence of amino acids at the C-terminal region (approximately 22–28 residues) (Figure 1), the monomer form of IfDPEase had the lowest molecular weight compared to other monomer forms of DPEases and ketose 3-epimerases, which were approximately 32–34 kDa [1,4].

After comparing the IfDPEase primary sequence with the sequences in the current databases of AllergenOnline, https://www.ddg-pharmfac.net/AllerTOP/ (accessed on 14 October 2022) and http://crdd.osdd.net/raghava/algpred/ (accessed on 14 October 2022), IfDPEase was found to be an allergen-safe protein for humans (Figure S3).

### 2.3. pI Values of IfDPEase and Other Ketose 3-Epimerases

The prediction of the pI values of IfDPEase and other ketose 3-epimerases from various microbial nucleotide sequencings was performed using the Expasy online application https://web.expasy.org/compute_pi/ (accessed on 24 September 2022) using all amino acids of each enzyme. As shown in Table 1, the pI value of IfDPEase (4.95) was the lowest compared to those of other ketose 3-epimerase members, which ranged from 4.98 to 5.94. These results suggest that IfDPEase may contain more acidic amino acids (glutamic and aspartic acids) on the surface of the enzyme structure than other ketose 3-epimerase members. Table 1 shows that 85.00% of aspartic and glutamic acid residues were detected on the surface of IfDPEase, whereas other ketose 3-epimerases were in the range of 63.15–82.60%.

### 2.4. Substrate Specificity

**Figure 2:**
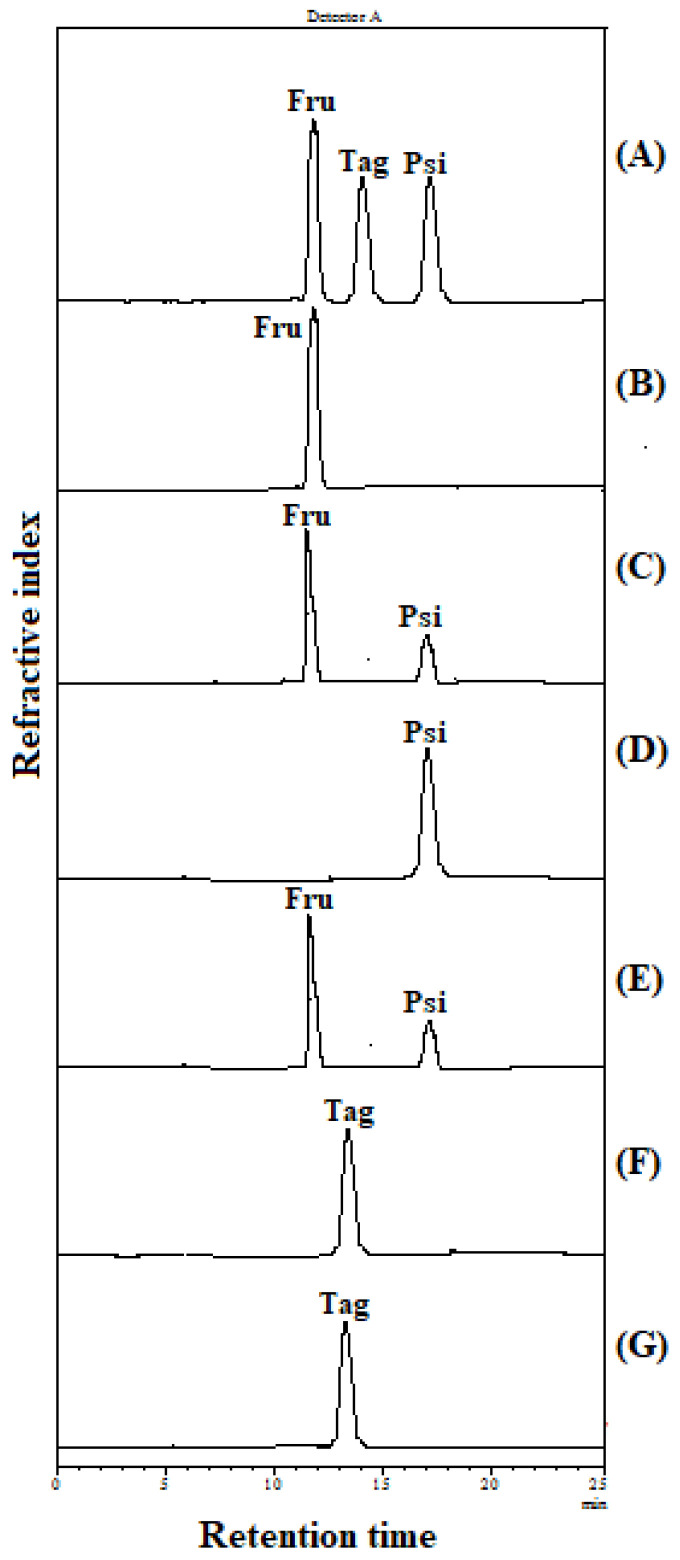
HPLC analysis profiles of IfDPEase (5 µM) on 10 mg/mL of D-fructose, D-psicose, and D-tagatose with 1.0 mM Mn^2+^ at 50 °C and pH 7.5 (50 mM Tris-HCl buffer) for 5 min. (**A**) Standards, D-fructose, D-tagatose, and D-psicose; (**B**,**D**,**F**) each substrate with denatured enzyme (**C**,**E**,**G**); IfDPEase reaction to D-fructose, D-psicose, and D-tagatose, respectively.

Ketose 3-epimerases from various microbial sources, including *C. bolteae*, *Dorea* sp. CAG317, *Ruminococcus* sp., *A. tumefaciens*, *C. cellulolyticum* H10, *B. produca*, *P. cichorii*, *C. fortuita*, and *R. sphaeroides*, exhibited broad substrate specificity on D-fructose, D-psicose, and D-tagatose (Table 3). As shown in Figure 2 and Table 3, IfDPEase showed a reversible epimerization reaction only toward D-fructose (67.62 U mg^−1^) and D-psicose (96.35 U mg^−1^) but could not catalyze D-tagatose. Thus, IfDPEase is more specific to the substrate than other ketose 3-epimerase members. The reaction scheme for the catalysis of keto sugars by IfDPEase is shown in Figure S4.

Most ketose 3-epimerases, including IfDPEase, prefer to catalyze D-psicose over D-fructose, possibly due to the formation of more hydrogen bonds between D-psicose and the enzyme [11]. However, the epimerization reaction ratio between D-fructose and D-psicose for IfDPEase (0.70) was higher compared to those of most other 3-epimerase members (0.33–0.66) except for the DFEase from *R. sphaeroides* (1.81), which prefers to catalyze D-fructose over D-psicose (Table 3).

### 2.5. Enzyme Kinetics

The Michaelis–Menten kinetic parameters of IfDPEase were measured under optimal conditions (1.0 mM Mn^2+^ at 50 °C, pH 7.5) against D-fructose. The affinity (*K*_m_), the turnover number (*k*_cat_), and the catalytic efficiency (*k*_cat_/*K*_m_) of the IfDPEase were 21.31 mM, 12.82 s^−1^, and 0.60 s^−1^ mM^−1^, respectively. Patel et al. (2021) [2] reported that the *K*_m_, *k*_cat_, and *k*_cat_/*K*_m_ of ketose 3-epimerase members toward D-fructose ranged from of 24.00–549.00 mM, 5.80–1059.55 (s^−1^), and 0.11–3.31 mM^−1^ s^−1^, respectively. Thus, the *k*_cat_ and *k*_cat_/*K*_m_ values of IfDPEase were in the same range as those reported previously. However, the *K*_m_ value of IfDPEase was the lowest compared to those of other ketose 3-epimerase members.

### 2.6. Effects of pH, Temperature, and Metal Ions on IfDPEase Activity

The IfDPEase showed high activity between pH 7.0 and 8.0, with an optimal pH of 7.5, while enzymatic activities of above 96% and 91% were detected at pH 8.0 and 6.0, respectively (Figure S5A). The optimum temperature was 50 °C, whereas at 40 and 60 °C, the enzymatic activity was greater than 80% and 98%, respectively. The IfDPEase remained active between 70 and 80 °C, with a relative activity greater than 50% (Figure S5B). The IfDPEase displayed maximum activity under optimal conditions (pH 7.5 and 50 °C) in the presence of 1.0 mM Mn^2+^ (100%), followed by Co^2+^ (60.01%), Ca^2+^ (16.98%), Na^+^ (16.37%), Mg^2+^ (10.09%), and K^+^ (8.55%), while in the presence of 1.0 mM ethylenediaminetetraacetic acid (EDTA), the enzymatic activity was 1.53% (Figure S6). This result indicates that IfDPEase is a metal-dependent enzyme.

Patel et al. (2021) [2] reported that optimal conditions of the characterized ketose 3-epimerase members are found in a pH range of pH 6.0 to 9.0 and at temperatures from 40 to 80 °C, and that Mn^2+^ and/or Co^2+^ are required for their enzymatic activities. Thus, the optimal pH and temperature for IfDPEase activity were in the same range as previously reported, whereas the metal ions required for enzymatic activity were similar to those required by other ketose 3-epimerase members.

### 2.7. Effect of Sodium Chloride on IfDPEase Activity

**Figure 3:**
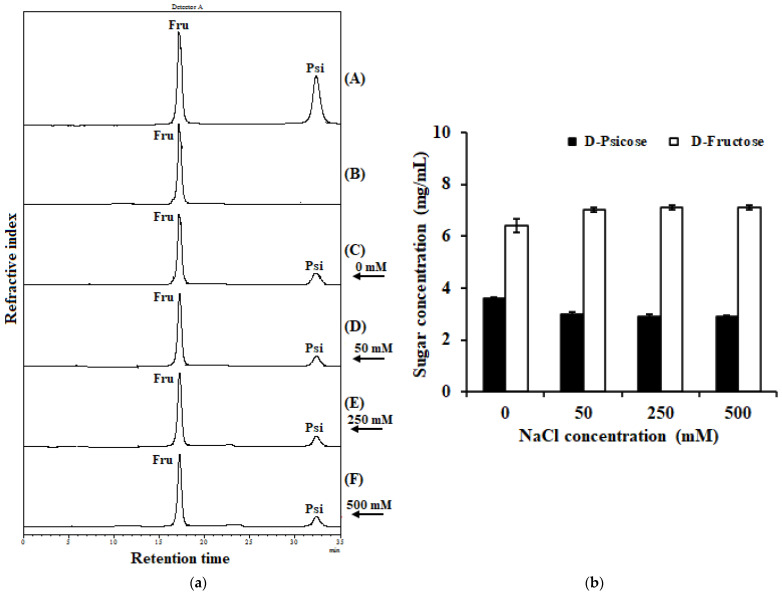
Effect of sodium chloride concentration on IfDPEase activity. (**a**) HPLC analysis profiles of reactions contained 0–500 mM NaCl. The reaction was determined for D-fructose (10 mg/mL) with 5 µM of the purified enzyme containing 1.0 mM Mn^2+^ at 50 °C and pH 7.5 (50 mM Tris-HCl buffer) for 5 min; (**A**) standard D-fructose and D-psicose, (**B**) the reaction with denatured enzyme, (**C**) the reaction without NaCl, (**D**–**F**) the reaction under 50, 250, and 500 mM NaCl, respectively. (**b**) Changing concentrations of D-fructose and D-psicose after incubation under various concentrations of NaCl. Values are the mean of three replications ± standard deviation.

Halophilic enzymes should be active and stable at high levels of salinity. To explain its tolerance to NaCl (a salt commonly used in food), IfDPEase (5 µM) was incubated at various concentrations of NaCl (0–500 mM) under optimum conditions (1.0 mM Mn^2+^, pH 7.5, and 50 °C). Although IfDPEase had the highest activity on D-fructose (10 mg/mL) in the absence of NaCl (obtained D-psicose 3.61 mg/mL), the enzyme activity remained greater than 2.90 mg/mL across all NaCl concentration ranges up to 500 mM (Figure 3). The results confirmed that this enzyme works well at high NaCl concentrations. We noted that at 500 mM NaCl, the enzyme did not precipitate or denature during the reaction.

### 2.8. Conversion of D-Fructose in Coconut Water into D-Psicose by IfDPEase

**Figure 4:**
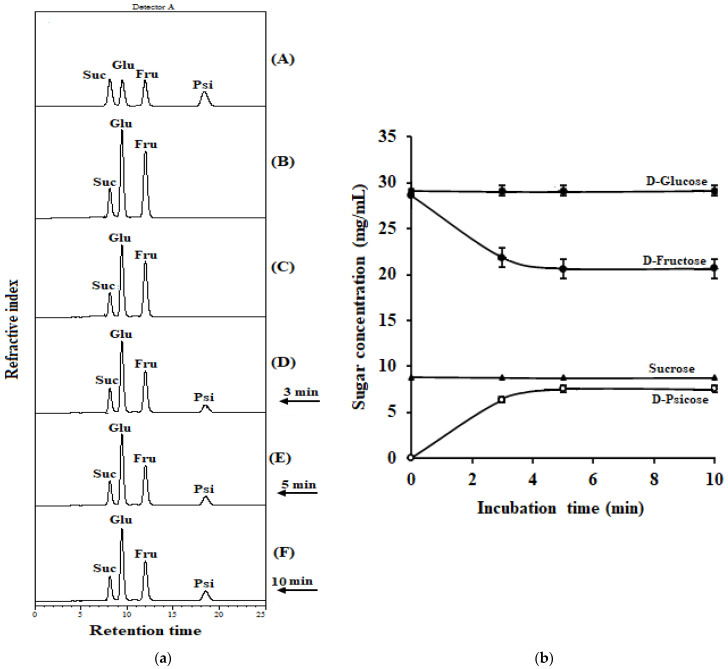
Conversion of D-fructose in coconut water into D-psicose by IfDPEase (15 µM). (**a**) HPLC analysis profile of the IfDPEase reaction to coconut water; (**A**) standard sucrose, D-glucose, D-fructose, and D-psicose; (**B**) coconut water without enzyme; (**C**) the reaction with the denatured enzyme; (**D**–**F**) the reaction at 3, 5, and 10 min, respectively. (**b**) Concentration of sugars changed during the reaction of IfDPEase with coconut water.

Santoso et al. (1996) [21] reported that coconut water contains D-fructose, D-glucose, and sucrose but not maltose and xylose. In this study, the fresh coconut water consisted of D-fructose (28.17 mg/mL), D-glucose (29.14 mg/mL), and sucrose (8.77 mg/mL) (Figure 4), and it had a pH of 6.5 at 25 °C. Moreover, the coconut water contained 1.12 mM manganese ions [12]. These conditions are suitable for IfDPEase activity. Thus, IfDPEase was used with fresh coconut water without adjusting any ingredients. After incubation at 50 °C for 3, 5, and 10 min, 6.36, 7.54, and 7.59 mg/mL of D-psicose were obtained from the D-fructose, respectively (Figure 4). On the other hand, the amounts of D-glucose and sucrose in the coconut water were constant. These two sugars cannot be catalyzed by the IfDPEase. After incubation at 50 °C for 5 min, IfDPEase was able to convert the D-fructose in coconut water into D-psicose, yielding approximately 26.77%.

## 3. Discussion

As a family of enzymes that can catalyze the C3 epimerization of various ketohexoses, DPEases particularly convert D-fructose into the rare, low-calorie sweetener D-psicose [2]. Although several ketose 3-epimerases, including DPEase, have been reported, most of them are produced by microorganisms growing under moderate conditions [1]. Therefore, ketose 3-epimerases are restricted for use under extreme conditions, especially at high salinity. Based on our knowledge, there have not been any reports of ketose 3-epimerases from extremely halophilic bacteria. Thus, this is the first report on the production of IfDPEase from an extremely halophilic, anaerobic bacterium.

Ketose 3-epimerases from many microorganisms exhibit a reversible epimerization reaction at the C3 positions of D-psicose (*cis*-ketose) and D-fructose (*trans*-ketose). In addition, these enzymes also show an epimerization reaction toward D-tagatose and L-sorbose. However, the activity of these enzymes is very low on L-sorbose due to their undetectable catalytic activity, whereas all ketose 3-epimerases prefer to catalyze D-tagatose over L-sorbose [3]. The specific activities of these enzymes toward D-fructose, D-psicose, and D-tagatose are shown in Table 3. The IfDPEase from the halophilic, anaerobic bacterium catalyzed only the conversion of D-fructose and D-psicose but not the conversion of D-tagatose (Figure 2). Considering the structures of D-psicose, D-fructose, and D-tagatose (Figure S4), D-psicose is an epimer of D-fructose at the C-3 position, D-tagatose is the *trans*-ketose epimer at the C-4 position of D-fructose (*cis*-ketose), and the C-4 position of D-psicose is *cis*-ketose. Therefore, unlike other members, IfDPEase can specifically epimerize ketohexoses at the C3 position, and the *cis*-ketose form at the C-4 position of ketohexoses is important for the function of this enzyme.

The enzymatic activity of most ketose 3-epimerases on D-psicose showed a higher level of activity than those on D-fructose (Table 3). Since IfDPEase is a true D-psicose 3-epimerase, it shows activity only against D-fructose and D-psicose. The epimerization reaction ratio between D-fructose and D-psicose for IfDPEase (0.70 times) was higher than the ratio for most other ketose 3-epimerases, such as *B. produca* (0.33 times), *P. cichorii* (0.33 times)*, C. cellulolyticum* (0.48 times), *A. tumefaciens* (0.51 times), *Ruminococcus* sp. 0.56 times), *C. fortuita* (0.60 times), *Dorea* sp. CAG317 (0.61 times), and *C. bolteae* (0.66 times) (Table 3). This result may be due to the fact that the *K*_m_ value (21.31 mM) of IfDPEase on D-fructose was lower than those of other ketose 3-epimerases (24.00–549.00 mM) [2], resulting in a better binding to D-fructose. Phakeenuya et al. (2020) [22] reported that when comparing the activity of a multifunctional enzyme (broad substrate specific enzyme) with that of other enzymes from the same enzyme family, the multifunctional enzyme had a lower enzyme activity compared to less-functional enzymes (more specific enzymes). In addition, the binding subsite residues, Gly218 and Cys6 of IfDPEase, which may be involved in interactions with the O-1 and O-6 positions of D-fructose substrate, respectively, differed from those of other ketose 3-epimerases (Table 2). Differences in amino acid residues in the binding subsites within active sites of these enzymes likely affect their function, and this may contribute to the increased efficiency of IfDPEase in the conversion of D-fructose into D-psicose.

We noted that some amino acid residues on the active site of *R. sphaeroides* DFEase, which prefer to catalyze D-fructose rather D-psicose (different from most ketose 3-epimerases) (Table 3), were also different from those of other ketose 3-epimerases (Table 2). One of the metal-coordinating site residues of this enzyme is glutamine, while the other ketose 3-epimerase member is histidine. On the other hand, the substrate-binding subsite residue that interacts with the O-4 position of D-fructose of this enzyme is glutamine, while the other ketose 3-epimerase member is histidine; the substrate-binding subsite residue at the O-6 of this enzyme is isoleucine, whereas the other 3-epimerases are cysteine, tyrosine, or phenylalanine.

Under optimum conditions (5 µM purified enzyme, 10 mg/mL D-fructose, 1.0 mM Mn^2+^, and pH 7.5 at 50 °C for 5 min), IfDPEase converted D-fructose into 3.61 mg/mL of D-psicose, yielding 36.1% (Figure 3B). The DPEase from *A. tumefaciens* produced 32.9% D-psicose from D-fructose under optimum conditions (pH 8.0 at 50 °C for 100 min) [14], whereas the DPEase from *C. cellulolyticum* H10 produced 29% D-psicose from D-fructose at 55 °C and pH 8.0 for 2 h [19], and the DPEse from *C. bolteae* was incubated with D-fructose at 55 °C and pH 6.5 for 200 min, yielding 28.8% D-psicose [13]. Further, the DFEase from *R. sphaeroides* obtained 17% D-psicose after incubation with D-fructose at 40 °C and pH 9.0 for 3 h [16]. Moreover, Tang et al. (2022) [4] reported that the conversion of D-fructose into D-psicose of ketose 3-epimerase enzymes ranges from 20% to 33%. Therefore, IfDPEase is a good alternative to the production of D-psicose from D-fructose due to its high yield in short reaction times; this contrasts with the findings of previous studies.

Halophilic enzymes tend to have excess acidic amino acids (glutamic and aspartic acids). The excess negative charges of these amino acids are localized at the surface of their enzyme structures, causing the low pI values of these enzymes, and the negative charges can protect Na^+^ ions from the environment. These enzymes are therefore active and highly stable under high salt concentrations and prevent precipitation during the reaction [6]. To date, the exploration of DPEase enzymes from halophilic bacteria is still limited. In a previous study, *I. fonsfrigidae* strain SP3-1 grew well at a high salt (sodium chloride) concentration of up to 30% (*w*/*v*) [23]. Thus, the DPEase produced from this bacterium should be tolerant toward high salt concentrations, in contrast to most enzymes from moderate microorganisms. Due to the large amount of acidic amino acid residues on the surface of this halophilic enzyme (Table 1), IfDPEase was active toward D-fructose (10 mg/mL) under high NaCl concentrations of up to 500 mM (Figure 3). In contrast, most non-halophilic enzymes/proteins are inactivated by low water activity and limited solvation under high salt concentrations, resulting in their denaturation, aggregation, and precipitation [6]. Until now, high concentrations of NaCl-resistant ketose 3-epimerases have not been reported. This property of IfDPEase makes it a suitable candidate as a processing aid for food products containing D-fructose and high concentrations of NaCl. Examples of food products containing D-fructose and sodium chloride that may be suitable for IfDPEase use include bakery products, confectionery products, corn snacks, dried fruits, seasoning sauces, and condiments [2,24].

*Iocasia* is a new genus of the family *Halanaerobiaceae*. Thus far, only one species with two strains, *Iocasia fonsfrigidae* strain NS-1 and strain SP3-1, has been reported [10]. Although these two strains have no confirmed toxicity, strain SP3-1 (this study) showed the highest similarity with another close-up genus in the family *Halanaerobiaceae*, namely, *Halocella cellulosilytica* DSM7362^T^, at 92.7%. Based on data from the Leibniz Institute DSMZ German Collection of Microorganisms and Cell Cultures GmbH, *H. cellulosilytica* is categorized as a biosafety level 1 microorganism https://www.dsmz.de/collection/catalogue/details/culture/DSM-7362 (accessed on 7 October 2022) with low risks for human health. Therefore, strain SP3-1 and the IfDPEase enzyme produced from this strain may be low-risk. However, prior to their use in food products, the toxicity of strain SP3-1 and IfDPEase must first be investigated, e.g., from a 13 week oral toxicity study in rats.

The world population is currently facing obesity and diabetes. Many high-calorie food products, especially those high in D-fructose, can cause issues [25]. These problems can be solved by consuming lower concentrations of D-fructose or replacing it with D-psicose, a healthy, low-calorie sweetener with a number of positive effects on human health [26]. D-psicose has gained considerable attention as a low-calorie sugar substitute, providing 70% sweetness when compared to sucrose [27]. Commercially available coconut water is a natural health drink with biological properties that are good for human health [8,9]; however, it contains 28.17 mg/mL of D-fructose. To reduce the D-fructose concentration and improve the quality of coconut water, IfDPEase was applied to fresh coconut water. The IfDPEase could convert the D-fructose in the coconut water into D-psicose (7.54 mg/mL), with a conversion yield of approximately 26.8% at 50 °C for 5 min without any adjustments. This is the first study on the ability of DPEase to improve the quality of coconut water. Improving the yield of D-psicose in coconut water may be achieved by increasing the concentration of IfDPEase in the reaction. Site-directed mutagenesis can also be used to improve the efficiency of IfDPEase in converting D-fructose into D-psicose.

## 4. Materials and Methods

### 4.1. Strains and Plasmid

The *I. fonsfrigidae* strain SP3-1 (formerly named *Halocella* sp. strain SP3-1) [23] is deposited at the Thailand Institute of Scientific and Technological Research Culture Collection (TISTR) and the Korean Collection for Type Cultures (KCTC) under the accession numbers TISTR.2992 and KCTC 25333, respectively. In addition, the *IfDPEase* gene of strain SP3-1 is deposited in GenBank with under the accession number OP035404. Plasmid pET-22b(+) (Novagen, Darmstadt, Germany) was used as the cloning and expression vector; it provides a protein containing a six-His tag at its C-terminus. The host cells for the recombinant plasmid were *E. coli* DH5α (Takara Bio, Shiga, Japan), while *E. coli* BL21 (DE3) (Novagen, Darmstadt, Germany) was used as the host for gene expression.

### 4.2. Chemicals and Reagents

D-Fructose, D-glucose, and sucrose were purchased from Sigma-Aldrich (St. Louis, MO, USA), and D-psicose and D-tagatose were obtained from TCI (Tokyo, Japan). Other chemicals and reagents were of analytical grade. The fresh, young coconut was purchased from a fresh-food market in Thung Khru District, Bangkok, Thailand. The pH of the coconut water was 6.5 at room temperature (~25 °C) and was measured using a pH meter (Orion Model 420A, Vernon Hills, IL, USA).

The Bradford protein assay with a bovine serum albumin (BSA) protein standard was purchased from Bio-Rad (Hercules, CA, USA). The protein purification column, HisTrap^TM^ (Ni Sepharose High Performance), was obtained from Merck (Darmstadt, Germany). Isopropyl β-D-1-thiogalactopyranoside (IPTG), imidazole, and ampicillin were obtained from Sigma (St. Louis, MO, USA). The electrophoresis set, including reagents and chemicals, was obtained from Bio-Rad (Hercules, CA, USA). The restriction enzyme *Bam*H1, *Xho*1, and T4 DNA ligase were purchased from New England BioLabs and Vivantis (Selangor Darul Ehsan, Malaysia). Precision Plus Protein^TM^ All Blue Prestained Protein Standard, ranging from 10 to 250 kDa, was purchased from Bio-Rad (Hercules, CA, USA).

### 4.3. Alignment and Allergenic Protein Sequence Search

The protein sequences of IfDPEase and other characterized ketose 3-epimerase members from the NCBI database were evaluated using the Clustal Omega multiple sequence alignment https://www.ebi.ac.uk/Tools/msa/clustalo/ (accessed on 10 September 2022). The protein allergen for food safety was assessed by implementing allergenic predication tools such as AllerTOP https://www.ddg-pharmfac.net/AllerTOP/ (accessed on 14 October 2022), and the Algpred database http://crdd.osdd.net/raghava/algpred/ (accessed on 14 October 2022).

### 4.4. Phylogenetic Tree of IfDPEase with Other Characterized Members

The phylogenetic tree of IfDPEase and other characterized ketose 3-epimerase members was constructed using the neighbor-joining method and a bootstrap analysis of 1000 replicates in the MEGA 11 Program [28]. The evolutionary distances were calculated using the Jones–Taylor–Thornton (JJT) model + G. The protein sequence was evaluated for the identification of identical amino acid residues by performing a sequence comparison (Clustal Omega multiple sequence alignment) with previously characterized ketose 3-epimerase members.

### 4.5. Gene Manipulation, Expression, and Purification of Recombinant IfDPEase

The *DPEase* gene (locus_tag=D7D81_07110) was used to design the primers for amplifying the gene target, using the forward (5′-GCCGGATCCATGAAACTATCTATTTGTACTGAT-3′) and reverse (5′-CTCGAGCTATAAACCAAGCAGGTAATCATA-3′) primers that contain the restriction sites *Bam*H1 and *Xho*1 (underlined), respectively. The PCR amplification was performed using Q5 high-fidelity DNA polymerase (New England Biolabs, Ipswich, MA, USA). This encoded epimerase gene (*DPEase*) has a size of 807 bp. The amplified gene was purified using a DNA purification kit (Qiagen, Hilden, Germany) and digested with *Bam*H1 and *Xho*1 restriction enzymes. The gene fragment was cloned into the pET-22b(+) vector, and the recombinant plasmid, termed pET22b-DPEase, was transformed into the host *E. coli* BL21 (DE3) for expression. The transformant was cultivated in LB broth containing ampicillin (100 µg/mL) as a starter and incubated at 37 °C under 200 rpm until the OD_600_ reached ~0.6. Protein expression was induced with 1 mM IPTG, and the cultures were further incubated at 16 °C for 18 h. After cultivation, cells were harvested and disrupted by sonication. The disrupted cells were centrifuged at 8000× *g* at 4 °C for 20 min, and the cell-free protein extract was purified using a HisTrap HP column. The purity of the purified protein was assessed by SDS-PAGE. A native-PAGE was performed similarly to the SDS-PAGE, but SDS, β-mercaptoethanol, and heat were not required [29]. The protein concentration was determined with the Bradford assay, using BSA as a standard.

### 4.6. Enzyme Assay

The reaction mixture for the enzyme assay consisted of the purified enzyme (5 μM) and 10 mg/mL of D-fructose in 50 mM Tris-HCl buffer pH 7.5 and 1.0 mM MnCl_2_. After incubation at 50 °C for 5 min, the reaction was stopped by boiling at 100 °C for 10 min, and the D-psicose was then analyzed using high-performance liquid chromatography (HPLC) (Shimadzu, Kyoto, Japan) with an RID-10A refractive index detector (Shimadzu RID-10A, Kyoto, Japan). The samples were separated on an SP0810 carbohydrate column (Shodex, Tokyo, Japan). aMilli-Q filtered water (EMD Millipore, Bedford, MA, USA) at 85 °C and with a flow rate of 1 mL/min was used as a mobile phase. Both D-fructose and D-psicose were used as standards. One unit (U) of enzyme activity was defined as the amount of enzyme catalyzing the formation of 1 µmol of D-psicose from D-fructose per minute under the assay conditions. All assays were performed in triplicate.

### 4.7. Substrate Specificity of IfDPEase

The substrate specificity of IfDPEase was evaluated using three types of substrates (D-fructose, D-psicose, and D-tagatose). The reaction mixture consisted of the purified enzyme (5 μM), 10 mg/mL of each substrate, and 1.0 mM MnCl_2_ in 50 mM Tris-HCl buffer at pH 7.5. After incubation at 50 °C for 5 min, the decrease in the substrate was qualitatively determined by HPLC as previously described; D-fructose, D-psicose, and D-tagatose were used as standards. All assays were performed in triplicate.

### 4.8. Effects of pH, Temperature, and Metal Ions on IfDPEase Activity

The effect of pH on IfDPEase activity was measured using 5 μM of the purified enzyme, using D-fructose (10 mg/mL) as a substrate and 1.0 mM MnCl_2_ at 50 °C in 50 mM of a buffer with pH values ranging from 6.0 to 8.0 (sodium phosphate, pH 6.0–7.0; Tris–HCl buffer, pH 7.0–8.0) for 5 min. The effect of temperature on enzyme activity was investigated at temperatures ranging from 30 to 80 °C at pH 7.5 for 5 min. The effect of metal ions was determined at 50 °C in 50 mM Tris-HCl buffer at pH 7.5 which contained various divalent and monovalent metal ions, including Ca^2+^, Co^2+^, K^+^, Mg^2+^, Mn^2+^, and Na^+^ (all metal ions used chloride salts) with a final concentration of 1 mM. Enzyme activity was measured as described above. All assays were performed in triplicate.

### 4.9. Effect of Sodium Chloride on IfDPEase Activity

To measure the effect of NaCl on enzyme activity, various NaCl concentrations (0–500 mM) were used. The reaction mixture consisted of the purified enzyme (5 μM), 10 mg/mL of D-fructose, and 1.0 mM MnCl_2_ in 50 mM Tris-HCl buffer at pH 7.5 and 50 °C for 5 min. The D-Psicose was analyzed by HPLC with an RID-10A refractive index detector on a salt-resistant column, the Aminex HPX-87P carbohydrate column (Bio-Rad, Hercules, CA, USA), operated at 85 °C with Milli-Q filtered water (EMD Millipore, Bedford, MA, USA) at a flow rate 0.6 mL/min as a mobile phase. All assays were performed in triplicate.

### 4.10. Prediction of the Isoelectric Point (pI) and Acidic Amino Acids on the Surface of Ketose 3-Epimerase Structures

The prediction of the pI values of ketose 3-epimerases from various microorganisms was performed using the Expasy online application https://web.expasy.org/compute_pi/ (accessed on 24 September 2022) through the total amino acids of each ketose 3-epimerase. Acidic amino acids (aspartic and glutamic acids) located on the surface of the ketose 3-epimerase structures of various microorganisms were performed using the NetsurfP-3.0 https://dtu.biolib.com/NetSurfP-3/ (accessed on 18 October 2022) [30] and the PredictProtein https://login.predictprotein.org/ (accessed on 18 October 2022) online tools by calculating the aspartic and glutamic acids on the surface of each ketose 3-epimerase.

### 4.11. Determination of Enzyme Kinetics

Kinetic parameters were determined by incubating the enzymes (5 µM) with different amounts of D-fructose (1 to 10 mM) containing 1.0 mM of Mn^2+^ in 50 mM Tris-HCl buffer at 50 °C and pH 7.5 for 5 min. Kinetic constant values were determined from Lineweaver–Burk plots. All assays were performed in triplicate.

### 4.12. Application of IfDPEase to Coconut Water

The purified IfDPEase was applied directly to fresh coconut water without adding any buffers or metal ions. The IfDPEase (15 µM) was added to coconut water with a final volume of 450 µL and incubated at 50 °C for 5 min. After incubation, aliquots of the reactant were removed and boiled at 100 °C for 10 min. The reaction mixture was analyzed by HPLC using an SP0810 carbohydrate column as described above, using D-glucose, D-fructose, D-psicose, and sucrose as standards. All assays were performed in triplicate.

## 5. Conclusions

IfDPEase is the first halophilic, anaerobic bacterial D-psicose 3-epimerase to be characterized and reported. This enzyme is active under high concentrations of NaCl and demonstrated a different substrate specific activity compared to other ketose 3-epimerases. Moreover, it can be used directly to improve the quality of natural products, such as coconut water, by converting D-fructose into D-psicose without any ingredient adjustments. Therefore, IfDPEase has great potential as a producer of D-psicose for food applications.

## Figures and Tables

**Table 1:** Total number of acidic amino acids residues (aspartic and glutamic acids), acidic amino acid residues on protein surfaces, percentage of aspartic and glutamic acids on protein surfaces, pI values, and % similarity of IfDPEase compared to other ketose 3-epimerases from various microorganisms. The analysis of aspartic and glutamic acids on the protein surface was obtained from the NetsurfP-3.0 https://dtu.biolib.com/NetSurfP-3/ (accessed on 18 October 2022) and the PredictProtein https://login.predictprotein.org/ (accessed on 18 October 2022) online tools. The pI values were calculated from Expasy https://web.expasy.org/compute_pi/ (accessed on 24 September 2022). The red letter is the IfDPEase, which showed the highest percentage of acidic amino acids on the protein surface, and the lowest pI value.

Source	Total Number of Aspartic and Glutamic Acids	Aspartic and Glutamic Acids on the Protein Surface (NetsurfP-3.0)	Aspartic and Glutamic Acids on the Protein Surface (PredictProtein)	Percentage of Aspartic and Glutamic Acids on the Protein Surface (%)	pI	Similarity of IfDPEase Compared to Other 3-Epimerases (%)
*Iocasia fonsfrigidae* strain SP3-1	40 from 269 aa	34	34	85.00	4.95	100
*Bacillus* sp. KCTC 13219	46 from 288 aa	37	37	80.43	4.98	24.32
*Clostridium cellulolyticum* H10	43 from 293 aa	35	35	81.39	5.41	23.55
*Agrobacterium* sp. ATCC 31749	37 from 289 aa	27	27	72.97	5.94	22.01
*Ruminococcus* sp.	43 from 291 aa	32	32	74.41	5.24	21.84
*Agrobacterium tumefaciens*	38 from 289 aa	24	24	63.15	5.88	21.24
*Rhodobacter sphaeroides*	40 from 295 aa	33	33	82.50	5.20	20.93
*Treponema primitia* ZAS-1	41 from 295 aa	31	31	75.60	5.93	20.69
*Clostridium. scindens* 35704	47 from 289 aa	35	35	74.46	5.14	20.46
*Pseudomonas cichorii*	46 from 290 aa	38	38	82.60	5.21	20.38
*Desmospora* sp. 8437	49 from 289 aa	38	38	77.55	5.24	20.08
*Caballeronia fortuita*	44 from 291 aa	33	33	75.00	5.19	20.00
*Clostridium bolteae*	49 from 301 aa	39	39	79.59	5.04	19.92
*Dorea* sp. CAG317	46 from 289 aa	34	34	73.91	4.99	18.92

aa—amino acids.

**Table 2:** Comparison of conserved amino acid residues in the active site of characterized ketose 3-epimerases from various microorganisms, including the catalytic site, metal-coordinating site, and substrate-binding subsites. The substrate-binding subsites revealed amino acid residues that formed hydrogen bonds with O-1 to O-6 of D-fructose compared with the *C. cellulolyticum* H-10 DPEase–D-fructose hydrogen bond interactions [12]. The red letters represent unique amino acids which differ from other ketose 3-epimerases.

Active Site	*I. fonsfrigidae*	*Bacillus* sp.	*C. cellulolyticum*	*C.* *boltae*	*A. tumefaciens*	*P.* *cichorii*	*C. fortuita*	*R. sphaeroides*
**Catalytic site**	Glu^153^	Glu^150^	Glu^150^	Glu^155^	Glu^150^	Glu^152^	Glu^152^	Glu^156^
	Glu^247^	Glu^244^	Glu^244^	Glu^256^	Glu^244^	Glu^246^	Glu^246^	Glu^250^
**Metal-coordinating site**	Glu^153^	Glu^150^	Glu^150^	Glu^162^	Glu^150^	Glu^152^	Glu^152^	Glu^156^
	Asp^186^	Asp^183^	Asp^183^	Asp^195^	Asp^183^	Asp^185^	Asp^185^	Asp^189^
	His^212^	His^209^	His^209^	His^221^	His^209^	His^211^	His^211^	Gln^215^
	Glu^247^	Glu^244^	Glu^244^	Glu^256^	Glu^244^	Glu^246^	Glu^246^	Glu^250^
**Binding site on D-fructose for O-1**	Glu^153^	Glu^150^	Glu^150^	Glu^162^	Glu^150^	Glu^152^	Glu^152^	Glu^156^
	Gly^218^	Arg^215^	Arg^215^	Arg^227^	Arg^215^	Arg^217^	Arg^217^	Arg^221^
**O-2**	Asp^186^	Asp^183^	Asp^183^	Asp^195^	Asp^183^	Asp^185^	Asp^185^	Asp^189^
	His^189^	His^186^	His^186^	His^198^	His^186^	His^188^	His^188^	His^192^
	Glu^247^	Glu^244^	Glu^244^	Glu^256^	Glu^244^	Glu^246^	Glu^246^	Glu^250^
**O-3 OE1**	Glu^153^	Glu^150^	Glu^150^	Glu^162^	Glu^150^	Glu^152^	Glu^152^	Glu^156^
**OE2**	Glu^153^	Glu^150^	Glu^150^	Glu^162^	Glu^150^	Glu^152^	Glu^152^	Glu^156^
**O-4**	His^212^	His^209^	His^209^	His^221^	His^209^	His^211^	His^211^	Gln^215^
**O-6**	Cys^6^	Tyr^6^	Tyr^6^	Tyr^16^	Tyr^6^	Phe^7^	Phe^7^	Ile^8^
**Reference**	This study	[2]	[11]	[13]	[14]	[15]	[3]	[16]

**Table 3:** Comparison of specific activity of ketose 3-epimerases from various microorganisms.

Enzyme	Source	Specific Activity (U mg^−1^)			Epimerization Reaction Ratio (D-Fructose/D-Psicose)	Reference
		D-Fructose	D-Psicose	D-Tagatose		
DPEase	*I. fonsfrigidae* SP3-1	67.62 ± 2.10	96.35 ± 1.70	ND	0.70	This study
	*C. bolteae*	150.70	226.90	52.70	0.66	[13]
	*Dorea* sp. CAG317	803.00	1310.00	64.18	0.61	[17]
	*Ruminococcus* sp.	8.95	16.00	0.15	0.56	[18]
	*A. tumefaciens*	8.89	17.50	5.93	0.51	[14]
	*C. cellulolyticum* H10	287.00	595.40	29.20	0.48	[19]
	*B. produca*	1.76	5.27	3.76	0.33	[4]
DTEase	*P. cichorii*	4.00	12.00	20.00	0.33	[20]
	*C. fortuita*	270.00	450.00	801.00	0.60	[3]
DFEase	*R. sphaeroides*	380.70	209.90	230.80	1.81	[16]

ND—could not detected under the assayed conditions.

## Data Availability

The authors confirm that the data supporting the findings of this study are available within the article.

## Supplementary Materials

The following supporting information can be downloaded at: https://www.mdpi.com/article/10.3390/ijms24076394/s1.
