# Supplementary material for: A Novel D-Psicose 3-Epimerase from Halophilic, Anaerobic Iocasia fonsfrigidae and Its Application in Coconut Water
Source: Int J Mol Sci. 2023 Mar 29;24(7):6394. doi: 10.3390/ijms24076394 (PMC10094494; doi:10.3390/ijms24076394)
Supplement: Supplementary file 1 [file ijms-24-06394-s001.zip › ijms-2273849-supplementary.pdf]

Supplementary Materials

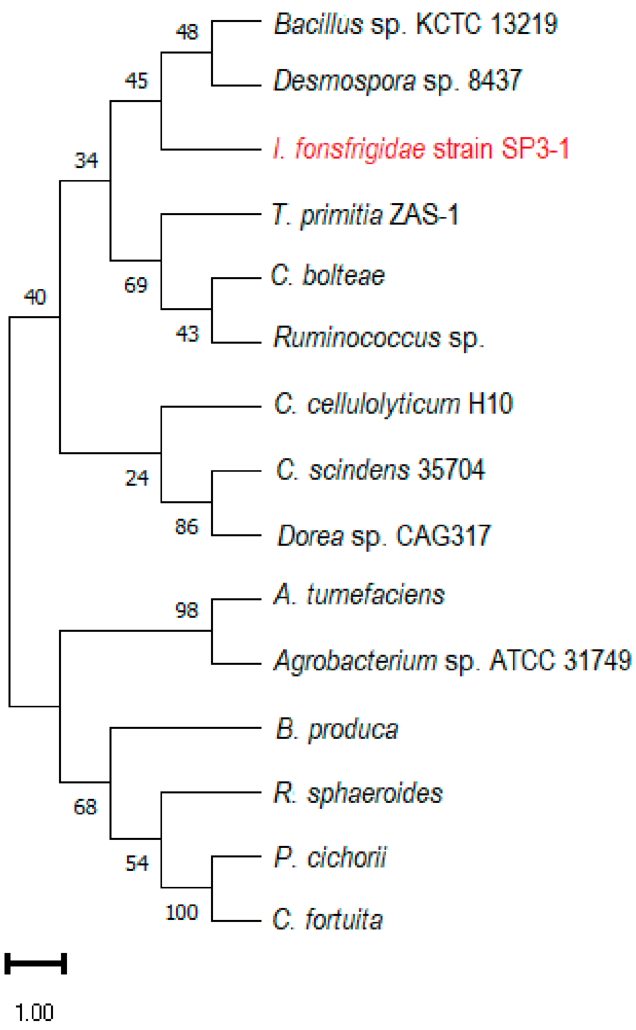

Figure S1. Phylogenetic tree analysis of IfDPEase with other characterized related members was constructed using protein sequences in MEGA 11 Program [26] by following Neighbor-Joining method with Jones-Taylor-Thornton (JJT) model + G (1000 Boostrap). The numbers at the branching points indicated the bootstrap values. The scale for branch length is shown below the tree. Red letter is DPEase from *I. fonsfrigidae* strain SP3-1 that has high relationship with DPEases from *Bacillus* sp. KCTC 13219, *Desmospora* sp. 8437, *Treponema primitia* ZAS-1, *Clostridium bolteae* 35704, *Ruminococcus* sp., *Clostridium cellulolyticum* H10, *Clostridium scindens* 35704, *Dorea* sp. CAG317, *Agrobacterium tumefaciens, Agrobacterium* sp. ATCC 31749, *Blautia produca*; DTEases from *Pseudomonas cichorii* and *Caballeronia fortuita*; and DFEase from *Rhodobacter sphaeroides*.

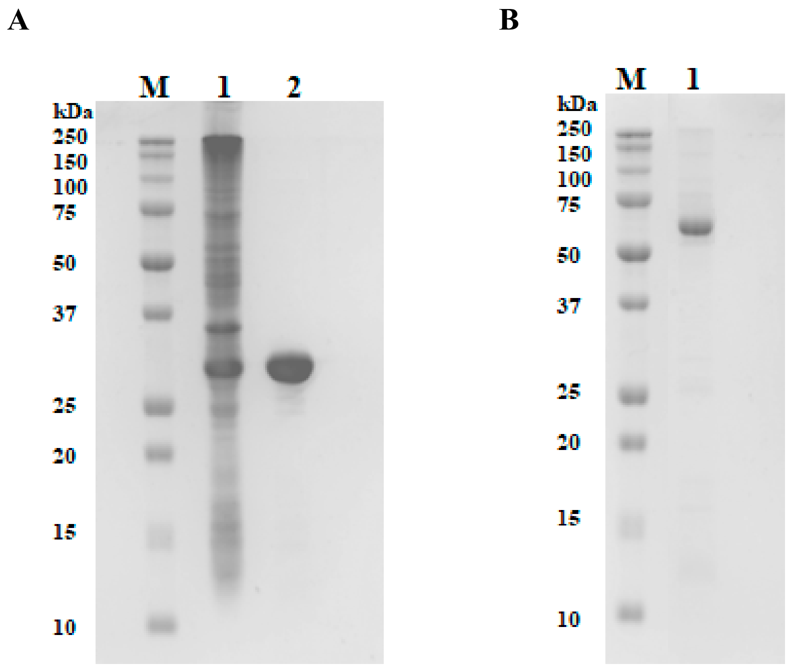

Figure S2. (**A**) SDS-PAGE of crude enzyme (lane 1) and purified IfDPEase (lane 2). (**B**) Native-PAGE of the purified IfDPEase (lane 1). Lane M is standard marker proteins.

A

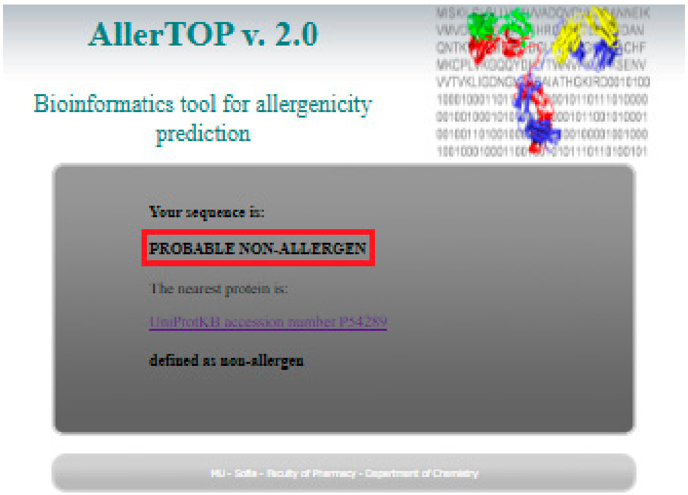

B

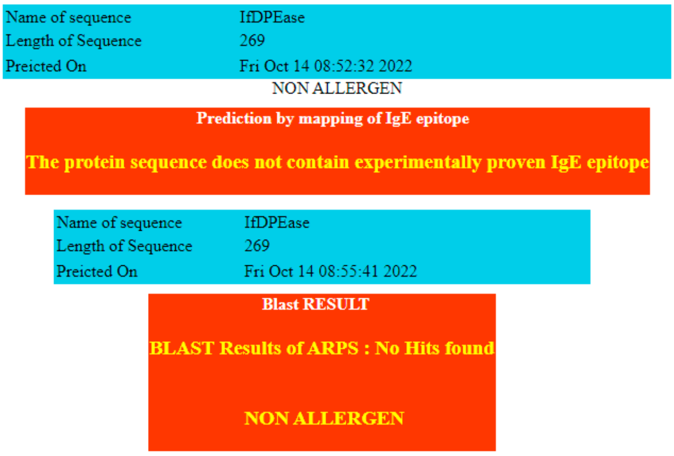

Figure S3. The protein allergen for food safety was performed. (**A**) Using the AllerTOP v.2.0 database by searching for full IfDPEase sequence compare with database (https://www.ddg-pharmfac.net/AllerTOP/). (**B**) Using the Algpred (http://crdd.osdd.net/raghava/algpred/) database by comparing sequence in UniProtKB and mapping of IgE epitope. Based on these results, IfDPEase was found to be non-allergenic.

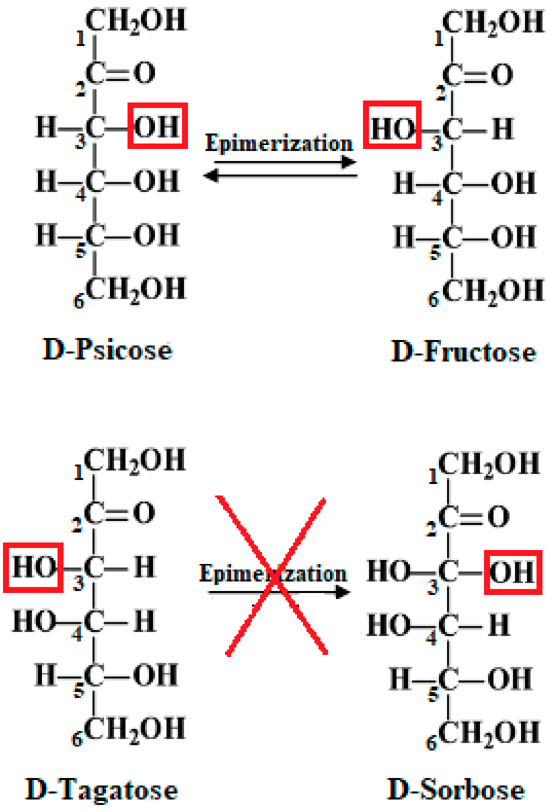

Figure S4. Reaction scheme for the catalysis of keto-sugars by IfDPEase

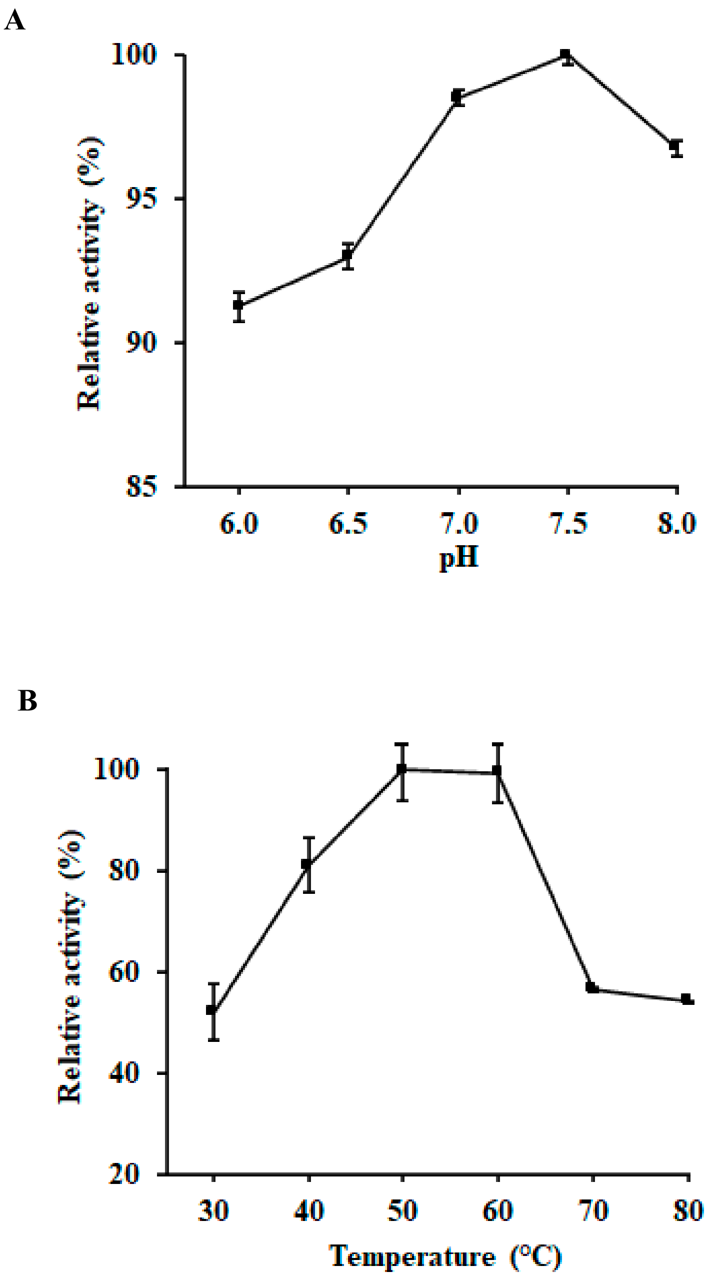

Figure S5. (**A**) Effect of pH on IfDPEase activity was determined by incubating 50 µM of purified enzyme with D-fructose (10 mg/mL) containing 1 mM of Mn^{2+} for 5 min in buffers with a pH range of 6.0-8.0 (sodium phosphate buffer, pH 6.0-7.0; Tris-HCl buffer, pH 7.0-8.0). The reaction was done at 50 °C for 5 min. The maximum pH activity of IfDPEase at pH 7.5 was considered as 100% relative activity. (**B**) Effect of temperature on IfDPEase activity was determined under the conditions described above.The reaction was done with a temperature range of 30 to 80 °C, pH

7.5 for 5 min. The maximum temperature activity of IfDPEase at 50 °C was considered as 100% relative activity. Values are mean of three replications ± standard deviation.

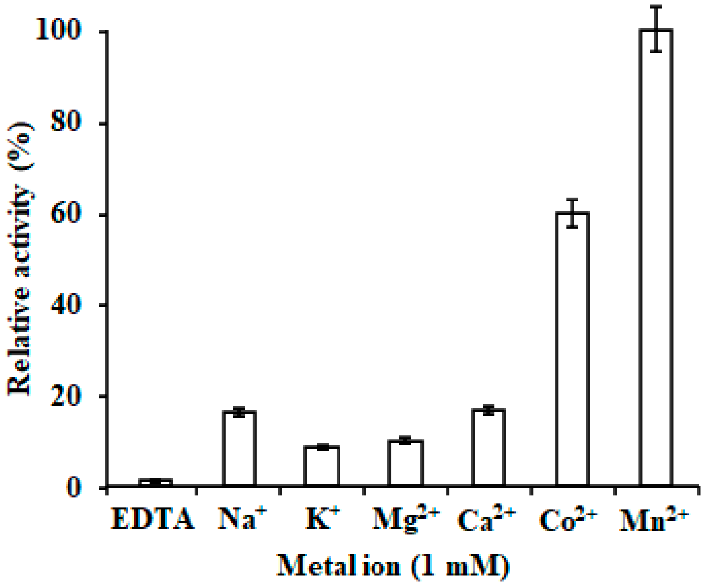

Figure S6. Effect of metal ions on IfDPEase activity. The reactions were done with D-fructose (10 mg/mL) and purified enzyme (5 µM) at 50 °C, pH 7.5 for 5 min with different metal ions. Metal ions used are Na^+, K^+, Mg^{2+}, Ca^{2+}, Co^{2+} and Mn^{2+}. The values are shown as percentages of the optimal activity with Mn^{2+}, taken as 100% relative activity. Values are the mean of three replications ± standard deviation.
